# Blood pressure and body fat percent in women with NMOSD

**DOI:** 10.1002/brb3.1350

**Published:** 2019-08-03

**Authors:** Xiaohong Chen, Rong Fan, Fuhua Peng, Jia Liu, Jing Huang, Zhigang Chen, Yong Chen, Ying Jiang

**Affiliations:** ^1^ Department of Neurology The Third Affiliated Hospital, Sun Yat‐sen University Guangdong China; ^2^ Department of Biostatistics, Epidemiology and Informatics University of Pennsylvania Philadelphia Pennsylvania

**Keywords:** blood pressure, body fat percent, body mass index, multiple sclerosis, neuromyelitis optica spectrum disorder

## Abstract

**Background:**

Hypertension is a prevalent and impactful comorbid condition among patients with multiple sclerosis (MS). High level of body mass index (BMI) is associated with the risk and poor outcomes of neuromyelitis optica spectrum disorder (NMOSD) in women. However, the clinical implication of blood pressure (BP) and body fat percent (BF%) based on the Clínica Universidad de Navarra‐Body Adiposity Estimator (CUN‐BAE) in NMOSD has not been investigated thus far.

**Methods:**

Case data were collected from 47 NMOSD and 28 MS patients at acute phase, 21 NMOSD and 25 MS patients at stable phase, and 68 age‐ and sex‐matched HCs. Four BP measures including systolic BP (SBP), diastolic BP (DBP), pulse pressure (PP), and mean arterial pressure (MAP); BMI; and BF% between NMOSD, MS, and healthy controls were determined.

**Results:**

Comparing NMOSD patients with MS patients, the former have significantly higher SBP (*p* < 0.001), DBP (*p* < 0.001), PP (*p* < 0.001), MAP (*p* < 0.001), BF% (*p* = 0.001), and BMI (*p* < 0.001) levels at acute phase after adjusting for age. Acute myelitis (OR 3.719, 95% CI 1.110–12.453) is more likely to occur in NMOSD patients with high BF% (≥30%) at acute phase. BF% was negatively correlated with 1/AQP4 titer in NMOSD at acute phase (*r* = −0.522, *p* = 0.004).

**Conclusions:**

Women with NMOSD are probably more prone to have an increased BP and fat mass compared to MS.

## 1 INTRODUCTION

Hypertension, which is one of the five leading causes of disability in the general population, is also a prevalent and impactful comorbid condition among patients with multiple sclerosis (MS) (Marrie et al., 2012). A meta‐analysis had reported that 18.6% of the MS population had hypertension (Marrie et al., 2015), and 3.73% of an incident MS cohort was found to develop hypertension over a maximum follow‐up of 30 years (Christiansen et al., 2010). Hypertension is linked to more rapid progression of ambulatory disability and an increased risk of cane use in patients with MS (Marrie et al., 2010). Pulse pressure has been associated with ambulatory functioning in patients with MS (Heffernan et al., 2011). A study also showed significantly lower systolic blood pressure (BP) readings in MS patients compared to non‐MS patients (Sternberg et al., 2013). A recent study has reported that hypertension was the most prevalent comorbidity in NMO and the prevalence of hypertension among highly active NMO cases was almost three times higher versus those in matched controls (Ajmera, Boscoe, Mauskopf, Candrilli, & Levy, 2018). Another study showed no differences in the prevalence of hypertension between NMOSD compared to control participants, while it was 30%–50% higher in MS participants compared to the controls (Saroufim, Zweig, Conway, & Briggs, 2018). However, no studies to date examined the BP including systolic BP (SBP), diastolic BP (DBP), pulse pressure (PP), and mean arterial pressure (MAP) in patients with neuromyelitis optica spectrum disorder (NMOSD).

Body mass index (BMI) is widely used for identifying obesity and assessing health risk associated with excess body fat. However, it cannot fully reflect body fat differences across sex, age, and race (Camhi et al., 2011; Rush, Freitas, & Plank, 2009) and classify individuals with high muscle mass as overweight or obesity (Witt & Bush, 2005). Therefore, new practical adiposity indices, such as the Clínica Universidad de Navarra‐Body Adiposity Estimator (CUN‐BAE), are suggested as more accurate estimators for determining body fat percent (BF%) (Gómez‐Ambrosi, Silva, Catalán, et al., 2012). CUN‐BAE, which is based on age, sex, and BMI (Gómez‐Ambrosi, Silva, Catalán, et al., 2012; Gómez‐Ambrosi, Silva, Galofré, et al., 2012), has showed the highest correlation with BF% compared with other anthropometric measures (Gómez‐Ambrosi, Silva, Catalán, et al., 2012). Several studies have demonstrated that higher BMI might be associated with an increased risk of MS, comorbid conditions, and disability progression (Hedstrom, Olsson, & Alfredsson, 2012; Marck, Neate, Taylor, Weiland, & Jelinek, 2016; Marrie, 2016; Munger, Chitnis, & Ascherio, 2009). Recently, a study has showed that low BMI could be associated with the risk and poor outcomes of NMOSD in women (Baek et al., 2018). However, it is still unknown about the clinical implication of BF% based on CUN‐BAE in NMOSD.

There are few reports assessing BP and BF% in individuals with NMOSD, although it is important given its impact on patients’ quality of life, adherence to treatment (especially steroids therapy), and prognosis. To the best of our knowledge, no specific studies have been focused on the BP and BF% based on CUN‐BAE in NMOSD and MS patients without treatment. To fill this research gap, we conducted the study to compare the BP and BF% between NMOSD and MS female patients, based on SBP, DBP, PP, MAP, BMI, and BF%. Establishing the prevalence and profile of BP and BF% in NMOSD remains of key importance, not only in understanding these conditions but also for informing the development of specialist services based on the needs of individuals with these conditions.

## 2 MATERIALS AND METHODS

### 2.1 Samples and study design

This is a prospective study approved by the Medical Ethics Committee of the Third Affiliated Hospital of Sun Yat‐sen University. All study participants have provided written consent for research and publication. All study participants were Chinese Han women.

We recruited 47 NMOSD female patients and 28 MS female patients at acute phase without steroids or other disease‐modifying immunosuppressive therapy in the three months prior to admission from the Department of Neurology and Multiple Sclerosis Research Center, the Third Affiliated Hospital of Sun Yat‐sen University, during November 2012 and November 2017. In addition, we also recruited 68 age‐ and sex‐matched healthy controls (HCs) from the Department of Medical Examination Center. Diagnosis criteria for NMOSD and MS patients were based on the 2015 international consensus diagnostic criteria (Wingerchuk et al., 2015) and the McDonald criteria 2010 (Polman et al., 2011), respectively. All data of these NMOSD and MS patients at acute phase were collected before treatment (methylprednisolone pulse therapy or IV immunoglobulin or immunosuppressive therapy). Patients were tested for antiaquaporin‐4 (AQP4) antibody using cell‐based assay from a commercial BIOCHIP kit (EUROIMMUN, Lübeck, Germany) (Long et al., 2012).

Data of 21 NMOSD patients with low‐dose glucocorticoid therapy (<10 mg prednisone or 8 mg methylprednisolone daily) and 25 MS patients without steroids or other disease‐modifying immunosuppressive therapy were collected at stable phase of disease. Patients who had experienced a relapse within the past month were considered to be in the acute phase, whereas those who were in a stable condition and had not experienced a clinical relapse for more than 1 month were considered to be in the stable phase. A relapse was defined as new or worsening CNS symptoms or signs that persisted for more than 24 hr. These patients included the patients who visited our hospital for the first time at the onset of the disease and the patients who visited the hospital at the relapse or stable stage. Our hospital, which is located on Guangzhou City, Guangdong Province, is a tertiary medical center and the major research unit focused on MS and NMOSD in China. Patients mainly come from South China, especially Guangdong Province, Guangxi Province, Hainan Province, Hunan Province, and Jiangxi Province, and the population base is more than 200 million people.

Serum NMO‐IgG was detected in 42 NMOSD patients at acute phase (42/47, 89.36%) (Table 1). According to the core clinical characteristics at acute phase, 22 (22/47, 46.81%) NMOSD patients had acute myelitis, 21 (21/47, 44.68%) patients had optic neuritis, and 4 (4/47, 8.51%) patients had other core clinical characteristics (acute brainstem syndrome or other intracranial lesions) (Table 4). All patients were scored using the Expanded Disability Status Scale (EDSS). In addition, the patient’s serum antinuclear antibody (ANA), anti‐Sjogren syndrome A (anti‐SSA) antibody, and anti‐Sjogren syndrome B (anti‐SSB) antibody were detected. The remaining clinical data collected were as follows: age at the time of disease onset, disease duration, and annualized relapsing rate (ARR). And no patients and HCs had obviously abnormal thyroid function and habit of smoking. Subjects presenting serious concomitant illnesses (i.e., cancer or hepatitis) or treatments (i.e., chemotherapy) possibly interfering with cardiovascular risk were excluded.

**Table 1:** Demographic characteristics of patients with NMOSD, MS at acute phases, and HCs

	NMOSD	MS	HCs	*P*	P*
Patient number	47	28	68		
Current age, median (IQR), y Onset age, median (IQR), y	34.00 (25.00, 48.00) 32.00 (23.00, 46.00)	29.00 (22.00, 38.00) 23.00 (19.25, 31.75)	36.00 (27.00, 41.75)	0.046^a^	0.032^b^ 0.005^b^
Serum NMO‐IgG, n (%)	89.36%	0%			
Disease duration, median (IQR), y	0.67 (0.25, 4.00)	1.67 (0.75,7.00)			0.043^b^
ARR, median (IQR)	1 (1.0, 2.0)	1 (0.5, 1.5)			0.091^b^
EDSS, median (IQR)	4.5 (3.0, 5.5)	3.0 (2.5, 4.0)			0.003^b^
Height (m), mean ± SD	1.59 ± 0.05	1.59 ± 0.05	1.58 ± 0.06	0.898^c^	0.732^b^
Weight (kg) (IQR)	53 (49, 58)	51. 5 (46.25, 54.875)	52 (49, 56.375)	0.119^a^	0.129^b^

Note

ARR, annualized relapsing rate; EDSS, Expanded Disability Status Scale.; HC, healthy control; IQR, interquartile range; MS, multiple sclerosis; NMO‐IgG, NMO‐immunoglobulin G; NMOSD, neuromyelitis optica spectrum disorder. *P*=refers to the comparison between NMOSD, MS, and HCs; *P* ^*^= refers to the comparison between NMOSD and MS.

a Kruskal–Wallis test.

b Mann–Whitney U test.

c ANOVA test.

### 2.2 Height and weight

Height and weight were measured to the nearest 0.1 cm and 0.1 kg, respectively. Participants wore clothing (uniform hospital clothing) and no shoes while assessing weight and height.

### 2.3 Body mass index (BMI)

BMI was calculated as weight in kilograms divided by height in meters squared. BMI was classified as follows: underweight, BMI<18.5 kg/m^2^; normal weight, BMI = 18.5 to 24.9 kg/m^2^; overweight, BMI = 25.0 to 29.9 kg/m^2^; and obese, BMI ≥30.0 kg/m^2^. The BMI of NMOSD and MS at acute phase is measured before the use of steroid treatment.

### 2.4 Body fat percent (BF%)

Body fat percent (BF%) is determining by the Clínica Universidad de Navarra‐Body Adiposity Estimator (CUN‐BAE) (Gómez‐Ambrosi, Silva, Catalán, et al., 2012; Gómez‐Ambrosi, Silva, Galofré, et al., 2012): BF% = −44.988+(0.503×age)+(10.689×gender)+(3.172×BMI)−(0.026×BMI2)+(0.181×BMI×gender)−(0.02×BMI×age)−(0.005×BMI^2^×gender)+(0.00021×BMI^2^×age) where male = 0 and female = 1 for sex and age in years. Cutoff points for BF% used for defining overweight (30.1%–34.9% for women) and obesity (≥35.0% for women) are those most frequently used in the literature (Bosy‐Westphal et al., 2006; Deurenberg et al., 2001; Okorodudu et al., 2010; Romero‐Corral et al., 2008). In this study, we defined 30% as cutoff points for BF% (high BF% (≥30%) and low BF% (<30%)).

### 2.5 Blood pressure

Resting SBP and DBP were measured with an automatic blood pressure cuff (Omron HEM‐7130, Omron Healthcare, Dalian, China) in a supine position (10 min rest) after 10 min of quiet rest by the average of two times. When the BP readings in these patients were recorded, these patients were not in situation with acute discomfort, anxiety, or sharp pain. And PP and MAP were calculated.

### 2.6 Statistical analyses

Age at the time of recruitment was compared across study cohorts (NMOSD, MS, and HCs) by using chi‐square test and Kruskal–Wallis test, respectively. Height was compared across study cohorts (NMOSD, MS, and HCs) by using ANOVA test. Characteristics were compared between NMOSD and MS patients using chi‐square (or Fisher’s exact) tests for categorical variables and 2‐sample *t* (or Mann–Whitney *U*) tests for continuous variables.

SBP, DBP, PP, MAP, BMI, and BF% were compared at both acute and stable phases using Kruskal–Wallis tests and multivariate linear regression analyses between the two study cohorts (NMOSD and MS), and at acute phase among the three study cohorts (NMOSD, MS, and HCs) while using Mann–Whitney *U* tests between every two cohorts. We also investigate the differences between high BF% (≥30%) and low BF% (<30%) and the clinical characteristics in acute NMOSD patients, adjusting for the demographic characteristics.

All statistical analyses were performed using SPSS 22. A two‐sided p<0.05 was considered statistically significant.

## 3 RESULTS

### 3.1 Baseline characteristics

Table 1 summarizes the baseline patient characteristics at acute phase. The NMOSD patients were relatively older than MS patients but comparable to the HCs, with median age of 34.00 and interquartile range (IQR) (25.00, 48.00), compared to 29.00 (22.00, 38.00) in MS patients and 36.00 (27.00, 41.75) in HCs. The difference was statistically significant (*p *= 0.046). The NMOSD patients also have shorter disease duration than MS patients (*p *= 0.043), with median and IQR of 0.67 (0.25, 4.00) years versus 1.67 (0.75, 7.00) years. The EDSS scores of NMOSD patients were significantly higher than MS patients, while the median and IQR of EDSS were 4.5 (3.0, 5.5) in NMOSD patients and 3.0 (2.5, 4.0) in MS patients (*p *= 0.003). Height, weight, and ARR were showed no significant differences between two patient groups.

#### 3.1.1 Comparisons among NMOSD, MS patients at acute phase, and HCs

BMI, BF%, SBP, DBP, PP, and MAP levels were compared between all of the NMOSD and MS patients at acute phase, and to the HCs, using Kruskal–Wallis tests, Mann–Whitney *U* test (unadjusted model), and multivariate linear regression analyses, adjusting for age (adjusted model). The results are presented in Tables 2 and 3.

**Table 2:** The comparisons of NMOSD and MS patients at acute and stable phases

Variable		NMOSD		MS		*P1*	*P2*	*P3*	*P4*
		Acute phase (*n* = 47)	Stable phase (*n* = 21)	Acute phase (*n* = 28)	Stable phase (*n* = 25)				
BMI, kg/m^2^	Adjusted^a^	21.72 ± 1.08	23.33 ± 1.33	20.36 ± 0.26	20.19 ± 0.46	<0.001	<0.001	<0.001	0.098
Body fat%	Adjusted^a^	29.63 ± 3.90	32.26 ± 4.31	26.46 ± 2.30	27.23 ± 1.62	<0.001	<0.001	0.016	0.169
Systolic BP (mmHg)	Adjusted^a^	115.5 ± 1.7	113.6 ± 0.84	111.1 ± 1.5	111.1 ± 6.0	<0.001	0.069	<0.001	0.984
Diastolic BP (mmHg)	Adjusted^a^	74.1 ± 1.7	72.2 ± 0.3	71.5 ± 1.5	70.7 ± 0.8	<0.001	<0.001	<0.001	0.024
Pulse pressure (mmHg)	Adjusted^a^	41.4 ± 0.01	41.3 ± 0.5	39.6 ± 0.02	40.4 ± 6.7	<0.001	0.531	0.245	0.554
Mean arterial pressure (mmHg)	Adjusted^a^	87.9 ± 1.7	86.0 ± 0.5	84.7 ± 1.5	84.2 ± 1.5	<0.001	<0.001	<0.001	0.203

Note

*P1*= refers to the comparison between acute phase in NMOSD and acute phase in MS. *P2*= refers to the comparison between stable phase in NMOSD and stable phase in MS. *P3*= refers to the comparison between acute phase and stable phase in NMOSD. *P4*= refers to the comparison between acute phase and stable phase in MS.

Abbreviations: BMI, body mass index; BP, blood pressure; IQR, interquartile range; MS, multiple sclerosis; NMOSD, neuromyelitis optica spectrum disorder; Pulse pressure, systolic BP‐diastolic BP.

a The adjusted model was calculated using a multivariable linear regression model, adjusted by age.

**Table 3:** The comparisons of NMOSD and MS patients at acute phase with HCs

Variable		Acute‐phase NMOSD	Acute‐phase MS	HCs	P*	*P1*	*P2*
BMI, kg/m^2^	Adjusted^a^	21.72 ± 1.08	20.36 ± 0.26	20.76 ± 0.79	<0.001	<0.001	0.011
Body fat%	Adjusted^a^	29.63 ± 3.90	26.46 ± 2.30	28.19 ± 3.23	<0.001	0.033	0.011
Systolic BP (mmHg)	Adjusted^a^	115.5 ± 1.7	111.1 ± 1.5	107.9 ± 7.0	<0.001	<0.001	0.018
Diastolic BP (mmHg)	Adjusted^a^	74.1 ± 1.7	71.5 ± 1.5	71.2 ± 2.6	<0.001	<0.001	0.610
Pulse pressure (mmHg)	Adjusted^a^	41.4 ± 0.01	39.6 ± 0.02	36.7 ± 4.5	<0.001	<0.001	0.001
Mean arterial pressure (mmHg)	Adjusted^a^	87.9 ± 1.7	84.7 ± 1.5	83.5 ± 4.0	<0.001	<0.001	0.114

Note

P*=according to Kruskal–Wallis test. *P1*=refers to the comparison between NMOSD and HCs. *P2*=refers to the comparison between MS and HCs.

Abbreviations: BMI, body mass index; BP, blood pressure; HC, healthy control; IQR, interquartile range; MS, multiple sclerosis; NMOSD, neuromyelitis optica spectrum disorder; Pulse pressure = systolic BP‐diastolic BP.

a The adjusted model was calculated using a multivariable linear regression model, adjusted by age.

In both the unadjusted and adjusted models, the mean BF% level of NMOSD patients at acute phase was significantly higher than MS patients at acute phase (adjusted *p *= 0.001). The adjusted mean and standard deviation were 29.63 ± 3.90 vs. 26.46 ± 2.30. After adjusting for age, higher BMI (*p* < 0.001), SBP (*p* < 0.001), DBP (*p* < 0.001), PP (*p* < 0.001), and MAP (*p* < 0.001) levels were observed in NMOSD patients compared to MS patients (Table 2).

In both the unadjusted and adjusted models, the mean SBP, PP, and MAP level of NMOSD patients at acute phase were significantly higher than HCs (adjusted *p *< 0.001 for SBP; adjusted *p *< 0.001 for PP; adjusted *p *< 0.001 for MAP, respectively). The adjusted mean and standard deviation were 115.5 ± 1.7 vs. 107.9 ± 7.0 mmHg for SBP, 41.4 ± 0.01 vs. 36.7 ± 4.5 mmHg for PP, and 87.9 ± 1.7 vs. 83.5 ± 4.0 mmHg for MAP, respectively. In the adjusted models, the BMI (*p *< 0.001), BF% (*p *= 0.033), and DBP (*p *< 0.001) levels of NMOSD patients were higher than HCs. In addition, lower BMI (*p *= 0.011) and BF% (*p *= 0.011), and higher SBP (*p *= 0.018) and PP (*p *= 0.001) levels were observed in MS patients at acute phase compared to HCs. We did not observe any statistically significant difference in DBP and MAP level between MS patients at acute phase and HCs using both the unadjusted and adjusted models (Table 3).

**Figure 1:**
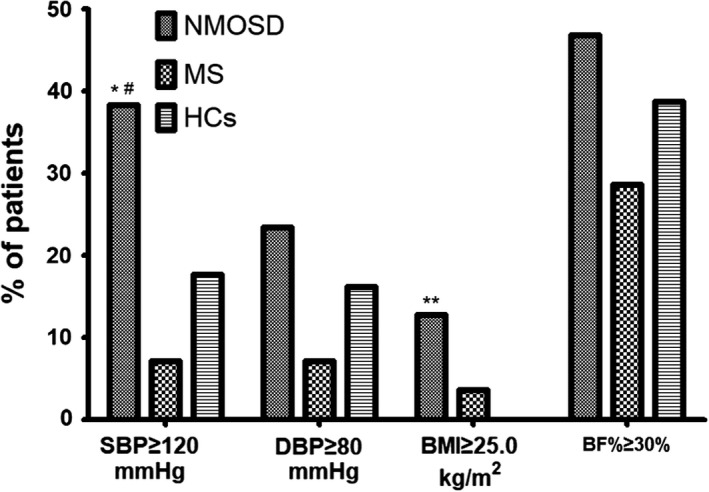
The percentages of NMOSD, MS, and healthy controls (HCs) with systolic BP (SBP) ≥ 120 mmHg, diastolic BP (DBP) ≥ 80 mmHg, body mass index (BMI) ≥ 25.0 kg/m^2^, and body fat percent (BF%) ≥30%.*refers to the comparison between NMOSD and HCs, p=0.013; **refers to the comparison between NMOSD and HCs, p=0.002; ^#^refers to the comparison between NMOSD and MS, *p* = 0.003

Among NMOSD patients at acute phase, 18/47 (38.3 %) of them presented with SBP readings above or equal to 120 mmHg, which is more than that in MS patients (2/28 (7.14%)) and in HCs (56/68 (17.6 %)). Besides, 11/47 (23.4 %) of them presented with DBP readings above or equal to 80 mmHg, which is also more than that in MS patients (2/28 (7.14 %)) and in HCs (11/68 (16.2 %)). BMI ≥25.0 kg/m^2^ was more likely to be found in NMOSD patients at acute phase (6/47 (12.8%)) than in MS patients at acute phase (1/28 (3.6 %)) and in HCs (0/68 (0.0%)). BF% ≥30% was found in 22/47 (46.8%) of NMOSD patients at acute phase, which is also significantly higher as compared to MS patients at acute phase (8/28 (28.6 %)) and HCs (27/68 (38.7%)) (Figure 1).

#### 3.1.2 Comparisons between NMOSD and MS patients at stable phase

In both the unadjusted and adjusted models, the mean BMI and BF% level of NMOSD patients at stable phase were significantly higher than MS patients at stable phase (adjusted *p *< 0.001 for BMI; adjusted *p *< 0.001 for BF%). After adjusting for age, higher DBP (*p* < 0.001) and MAP (*p* < 0.001) levels were observed in the NMOSD patients as compared to MS patients. We did not observe any statistically significant difference in SBP and PP level between NMOSD and MS patients using both the unadjusted and adjusted models (Table 2).

#### 3.1.3 Comparisons between acute and stable phases in NMOSD and MS patients

After adjusting for age, lower BMI (*p* < 0.001), and BF% (*p* = 0.016), higher SBP (*p* < 0.001), DBP (*p* < 0.001), and MAP (*p* < 0.001) levels were observed in acute phase compared to stable phase in NMOSD patients. We did not observe any statistically significant difference in PP level between acute phase and stable phase in NMOSD patients using both the unadjusted and adjusted models (Table 2).

In addition, statistically significant differences were not observed in BMI, BF%, SBP, PP, and MAP levels between acute phase and stable phase in MS patients using both the unadjusted and adjusted models. Only higher DBP (*p* < 0.024) level was observed in acute phase compared to stable phase in MS patients in the adjusted model (Table 2).

#### 3.1.4 Comparison between high BF% (≥30%) and low BF% (<30%) in NMOSD patients at acute phase

In this study, we defined 30% as cutoff points for BF% (high BF% (≥30%) and low BF% (<30%)). Except for BMI (*p* < 0.001), there were not any statistically significant differences in SBP, DBP, PP, MAP, EDSS scores, ARR, and 1/AQP4 titer levels between high BF% (≥30%) and low BF% (<30%) in NMOSD patients at acute phase. However, we found that acute myelitis (OR 3.719, 95% confidence interval (CI) 1.110–12.453, *p* = 0.03) was more likely to occur in NMOSD patients with high BF% (≥30%) at acute phase, but the other core clinical characteristics (acute brainstem syndrome or other intracranial lesions) (*p* = 0.05) were more likely to occur in NMOSD patients with low BF% (≥30%) at acute phase (Table 4).

**Table 4:** Comparison between NMOSD with high BF% (≥30%) and NMOSD with low BF% (<30%) at acute phase

Variables	NMOSD with high BF% (*n* = 22)	NMOSD with low BF% (*n* = 25)	*p*
Systolic BP (mmHg)	117.1 ± 13.0	114.0 ± 9.4	0.350
Diastolic BP (mmHg)	75.0 ± 8.3	73.2 ± 9.9	0.516
Pulse pressure (mmHg)	42.1 ± 10.8	40.8 ± 10.0	0.662
Mean arterial pressure (mmHg)	89.0 ± 8.7	86.8 ± 8.6	0.386
EDSS	4.5(3, 5.5)	4.5 (2.75, 5.5)	0.906^a^
1/AQP4 titer	100 (32, 100)^b^	100 (100, 100)^c^	0.368^a^
ARR	1.0 (0.72, 2.0)	2.0 (1.0, 2.0)	0.159^a^
Core clinical characteristics at acute phase, n			
Acute myelitis	14	8	0.03
Optic neuritis	8	13	0.282
Other d	0	4	0.05

Abbreviations: ARR, annualized relapsing rate; BMI, body mass index; BP, blood pressure; EDSS, Expanded Disability Status Scale; IQR, interquartile range; NMOSD, neuromyelitis optica spectrum disorder.

a Mann–Whitney U test.

b *n* = 13.

c *n* = 15.

d Refers to the core clinical characteristics except for acute myelitis and optic neuritis.

#### 3.1.5 Correlation between BF% and 1/AQP4 titer in NMOSD patients at acute phase

**Figure 2:**
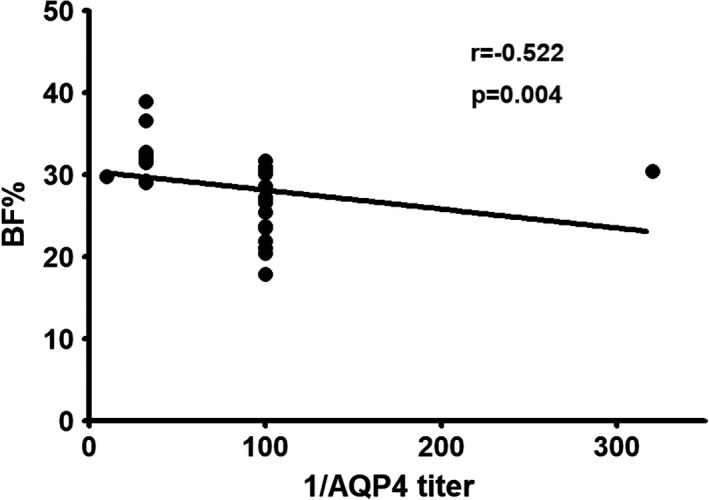
Scatter plot of the association between BF% and 1/AQP4 titer in women with NMOSD at acute phase. *N* = 28

Figure 2 showed BF% was negatively correlated with 1/AQP4 titer in NMOSD at acute phase (*r* = −0.522, *p* = 0.004). There were no correlations between BF% and EDSS scores, ARR, SBP, DBP, PP, or MAP. And there were no correlations between BMI and 1/AQP4 titer, EDSS scores, ARR, SBP, DBP, PP, or MAP in NMOSD patients at acute phase.

## 4 DISCUSSION

To our knowledge, this present study is the first to investigate BP and BF% in NMOSD and MS simultaneously. Based on this literature, it is clear that higher BP, including SBP, DBP, PP, and MAP, is a prevalent condition for NMOSD patients at acute phase. Among NMOSD patients at acute phase, 38.3% presented with the SBP readings ≥ 120 mmHg, as compared to 7.14% of MS patients at acute phase and to 17.6 % of age‐matched HCs, respectively. Following intergroup comparisons, the results also showed that NMOSD patients at acute phase exhibit higher BP (SBP, DBP, PP, and MAP) compared to MS. According to the results of this study, higher BP is probably more prevalent for NMOSD than MS.

Indeed, we have considered EDSS, BMI, or BF% as possible explanations for higher BP measures in NMOSD. Higher BMI or BF% has been reported to be associated with higher BP (Kobayashi et al., 2006; Mushengezi & Chillo, 2014), increasing the risk of hypertension (Jayedi, Rashidy‐Pour, Khorshidi, & Shab‐Bidar, 2018; Shuger, Sui, Church, Meriwether, & Blair, 2008) in general adult population. Our analyses identified that no significant correlations were observed between BMI or BF% and the four BP measures (SBP, DBP, PP, and MAP) in patients with NMOSD at acute phase. BP measures between NMOSD patients at acute phase with high BF% (≥30%) and with low BF% (<30%) were also calculated, while statistically significant differences were not found between two groups for SBP, DBP, PP, and MAP. This result suggested that BF% is not the likely explanation for higher BP readings in NMOSD. We also did not observe any significant correlations between EDSS and the four BP measures (SBP, DBP, PP, and MAP) in patients with NMOSD at acute phase. Similarly, the presence of hypertension was not associated with the EDSS scores in MS (Zhang et al., 2018). Interestingly, the present analyses indicated that acute myelitis more often occurred in NMOSD patients with high BF% (≥30%) at acute phase. The association between acute myelitis and high BF% should be researched further.

Glucocorticoid treatment is the recommended standard therapy for NMOSD, while high‐dose corticosteroids (typically intravenous methylprednisolone 1g/day for 3 to 5 days) are the mainstay of acute treatment and low‐dose corticosteroids are considered as part of the maintenance treatment of patients at stable phase (Bruscolini et al., 2018). However, the adverse effect on BP is dependent on the administered dose. Elevated BP had not been observed in those patients who accepted low‐dose corticosteroid therapy (Jackson, Beevers, & Myers, 1981; Panoulas et al., 2008). In our study, low‐dose glucocorticoid therapy was used on NMOSD at stable phase, and the readings of three BP measures (SBP, DBP, and MAP) of NMOSD at acute phase were still higher than those at stable phase, which probably suggested low‐dose glucocorticoid therapy at stable phase has little effect on the elevation of BP. This result further supports this conclusion that BP of NMOSD is higher at acute phase than at stable phase. And there was no corticosteroid therapy in MS patients at stable phase; SBP, PP, and MAP levels also were not altered compared to MS at acute phase.

The use of BMI is the most common way to assess overweight and obesity. Although it is frequently used as an indicator of BF%, it is highly imprecise in estimating body fat at an individual level (Gómez‐Ambrosi, Silva, Galofré, et al., 2012; Prentice & Jebb, 2001). Pilutti et al. (Pilutti & Motl, 2016) clearly showed that BMI significantly underestimates the amount of adipose tissue in MS. Our results also showed that among all the NMOSD patients at acute phase, 46.8% were overweight assessed by BF%, as well as 12.8% assessed by BMI, respectively. The prevalence of overweight by BMI value is higher among NMOSD at acute phase compared to HCs. Under the conditions of the present study in MS at acute phase, BM and BF% levels were not altered compared to MS at stable phase. However, the relatively higher prevalence of overweight and obesity in patients with NMOSD at stable phase compared to acute phase may be due to vitamin D deficiency, chronic corticosteroid therapy, and chronic inflammation.

In women with MS, there was no significant relation between BF% and EDSS (Lambert, Lee Archer, & Evans, 2002). The current study also did not find an association between BMI or BF% and ARR and EDSS in NMOSD. A recent study has showed that low BMI could be associated with the risk and poor outcomes of NMOSD in women (Baek et al., 2018). The prospective and longitudinal data are needed to further assess this association. Interestingly, the present result indicated that BF% was significantly related to 1/AQP4 titer in NMOSD patients at acute phase. The role of adipose tissue in the pathogenesis of MS has become subject of great interest (Guerrero‐García et al., 2016). It is believed that increased fat mass, as well as the elevated levels of adipokines, may be involved in the altered immune response and inflammatory processes in NMOSD. Therefore, increased fat mass may not only affect cardiovascular risk in NMOSD but also influence NMOSD progress.

### 4.1 Limitations

Our study has several limitations. Only a single ethnic population from a single center was evaluated, which can result in unintentional bias. The numbers of patients were relatively small. Besides, this study was performed only among female patients. The limited number of male patients with NMOSD could be responsible for it. Finally, the current study relied on CUN‐BAE for assessment of BF%. While DXA is a valid and reliable method for assessment of lean vs. fat mass, it is not able to assess more detailed aspects of body composition. Additional analysis of these fat stores using more advanced methods such as magnetic resonance imaging (MRI) may provide additional understanding of the impact of body composition on comorbidities in NMOSD.

## 5 CONCLUSIONS

Women with NMOSD are probably more prone to have an increased BP and fat mass compared to MS. Furthermore, for the individuals with NMOSD, there were no statistically significant relations between the body composition measures and EDSS score.

## CONFLICT OF INTEREST

All authors declare that there are no conflicts of interest.

## AUTHORS’ CONTRIBUTIONS

Y. J. contributed to the conception and design of this study. Y. J., X. C., R. F., F. P., J. L., and Z. C. collected and organized the data. Y. J., Y. C., X. C., R. F., J. H., and F. P. analyzed the data. Y. J., Y.C., Z. C., X. C., and R. F. drafted the manuscript. All the authors read and approved the final manuscript.

## ETHICS APPROVAL AND CONSENT TO PARTICIPATE

The study was conducted according to the principles expressed in the Declaration of Helsinki and approved by the Medical Ethics Committee of the Third Affiliated Hospital of Sun Yat‐sen University. All study participants gave written informed consent for research and publication.

## DATA AVAILABILITY STATEMENT

The datasets used and/or analyzed during the current study are available from the corresponding author on reasonable request.
